# Multifuntional Gold Nanoparticles for the SERS Detection of Pathogens Combined with a LAMP–in–Microdroplets Approach

**DOI:** 10.3390/ma13081934

**Published:** 2020-04-20

**Authors:** Alexandra Teixeira, Juan L. Paris, Foteini Roumani, Lorena Diéguez, Marta Prado, Begoña Espiña, Sara Abalde-Cela, Alejandro Garrido-Maestu, Laura Rodriguez-Lorenzo

**Affiliations:** International Iberian Nanotechnology Laboratory (INL), Avda Mestre José Veiga, 4715-310 Braga, Portugal; alexandra.teixeira@inl.int (A.T.); juan.paris@inl.int (J.L.P.); foteini.roumani@inl.int (F.R.); lorena.dieguez@inl.int (L.D.); marta.prado@inl.int (M.P.); Begona.Espina@inl.int (B.E.); sara.abalde@inl.int (S.A.-C.); alejandro.garrido@inl.int (A.G.-M.)

**Keywords:** gold nanoparticles, SERS, LAMP, microdroplets, glutathione, microfluidics, pathogens, *Listeria monocytogenes*

## Abstract

We developed a droplet-based optofluidic system for the detection of foodborne pathogens. Specifically, the loop-mediated isothermal amplification (LAMP) technique was combined with surface-enhanced Raman scattering (SERS), which offers an excellent method for DNA ultradetection. However, the direct SERS detection of DNA compromises the simplicity of data interpretation due to the variability of its SERS fingerprints. Therefore, we designed an indirect SERS detection method using multifunctional gold nanoparticles (AuNPs) based on the formation of pyrophosphate generated during the DNA amplification by LAMP. Towards this goal, we prepared multifunctional AuNPs involving three components with key roles: (1) thiolated poly(ethylene glycol) as stabilizing agent, (2) 1-naphthalenethiol as Raman reporter, and (3) glutathione as a bioinspired chelating agent of magnesium (II) ions. Thus, the variation in the SERS signal of 1-naphthalenethiol was controlled by the aggregation of AuNPs triggered by the complexation of pyrophosphate and glutathione with free magnesium ions. Using this strategy, we detected *Listeria monocytogenes*, not only in buffer, but also in a food matrix (i.e., ultra-high temperaturemilk) enabled by the massive production of hotspots as a result of the self-assemblies that enhanced the SERS signal. This allowed the development of a microdroplet-LAMP-SERS platform with isothermal amplification and real-time identification capabilities.

## 1. Introduction

In recent years, microfluidics technology has demonstrated increasing relevance in the development of analytical tools in different fields of research and applications [1,2]. Mainly, the miniaturization, automation, and standardization that microfluidics brings can be exploited to develop highly sensitive assays, ensuring reproducibility of results [3,4]. Microfluidic devices are very attractive in food safety analysis because they offer economic, portable, and disposable systems that can be used for on-site detection without the need of specific expertise when fully integrated. The implementation of point-of-care approaches for the detection of foodborne pathogens in the food chain is pivotal to food safety management [5]. The utilization of microfluidics, and more specifically microdroplets technology, a branch of microfluidics [6,7], may increase the options to achieve the implementation of point-of-care systems based on molecular techniques for the fast detection of pathogens in foodstuffs. 

Microdroplets can be used to compartmentalize all types of chemical and biological reactions in picoliter or nanoliter volumes at high-throughput with a fine control of the microdroplet contents [8,9,10,11,12]. As such, microdroplets have been used as laboratory flasks for the detection of pollutants, secreted proteins, bacteria and even single cells [8,13,14,15,16,17]. The molecular techniques are normally based on the detection of specific DNA or RNA target sequences using amplification processes, like the polymerase chain reaction (PCR) [18,19] and the most recent digital droplet polymerase chain reaction (ddPCR), that became commercially available in 2011. The ddPCR method is based on microdroplets technology, in which a single real-time PCR mixture is fractionated in droplets in real-time followed by DNA amplification, and downstream analysis [20]. Nevertheless, for some specific cases, the temperature cycles needed for the PCR may be detrimental for the aim of the experiment by damaging the involved reagents and/or biological elements [19]. Isothermal nucleic acid amplification technology, specifically loop-mediated isothermal amplification (LAMP), has been widely proposed as an alternative methodology because it exhibits good performance in ultrasensitive detection and biosensing, as compared to PCR [21,22]. The LAMP reaction requires a DNA polymerase with strand displacement activity and a set of four specially designed primers, termed inner and outer primers. The general DNA amplification reaction involves [23]:

(DNA)*_n_*_−1_ + dNTP → (DNA)*_n_* + P_2_O_7_^4^^−^ (1)

In DNA polymerization by DNA polymerase (1), pyrophosphate ions (P_2_O_7_^4^^−^) are released from deoxyribonucleosides triphosphates (dNTP) as a by-product. Different indirect detection formats based on the formation of pyrophosphates for visualizing LAMP products have been reported [24]. The large amount of pyrophosphate generated in LAMP reactions promotes the formation of a stable complex with magnesium ions, which are a component of the LAMP reaction buffer, generating an insoluble byproduct which can be measured in terms of turbidity [23]. Colorimetric LAMP assays using carboxyl acid-functionalized gold nanoparticles (AuNPs) have also been developed. The change of colour is controlled by the aggregation–disaggregation of AuNPs (e.g., from purple to red [25] or red to pink precipitate [26]) triggered by the complexation mechanism that involves carboxyl acid-contained molecules on Au surface, magnesium ions, and pyrophosphate ions generated in LAMP. This colorimetric LAMP assay was coupled with a microfluidic device using 11-mercaptoundecanoic acid-coated AuNPs for the detection of *Salmonella* spp. DNA [27]. Although LAMP has been successfully implemented in lab-on-chip systems [28], there are only some publications in which LAMP has been integrated with microdroplet technology [29,30]. However, the low volumes inherent to a microfluidic system force these strategies to be coupled to highly sensitive detection techniques. Surface-enhanced Raman scattering (SERS) spectroscopy has been successfully coupled with microfluidics and microdroplets for the development of sensitive, quantitative, and reproducible analytical platforms [31,32]. DNA detection by SERS can be performed either by label-free detection, or by indirect detection using a Raman reporter. Although label-free detection is simpler from an experimental point of view, data interpretation can be complicated in real samples because the signal may be indistinguishable and/or too low for reliable detection. Therefore, Raman reporters are often applied in the indirect detection of DNA, as they feature a much higher Raman cross section compared to DNA, or other biomolecules, and their SERS spectrum is easily recognizable [33]. In this line, Draz & Lu reported an integrated assay that combines LAMP and SERS for DNA indirect detection of *Salmonella enterica* using specifically designed AuNPs functionalized with Cy5/DNA probes [34]. 

Herein, we developed a bioinspired solution for the on-chip detection of *Listeria monocytogenes* by bringing together the advantages of molecular (LAMP), engineering (microfluidics), and spectroscopic (SERS) tools. *L. monocytogenes* is a well-known human pathogen and the causative agent of human listeriosis. In susceptible individuals (immunocompromised, elderly, children, and pregnant women), the disease can manifest with septicemia or meningoencephalitis [35]. Attending to the latest report published by the European Food Safety Authority and the European Centers for Disease Control and prevention, in 2018, more than 2500 cases of listeriosis were reported in Europe with a fatality rate of 15.6%, being one of the most serious foodborne diseases under the EU surveillance [36]. The fact that these figures have not significantly declined in the last few years highlights the need for seeking novel methodologies for the early detection of this pathogen.

In this work, we designed an indirect SERS detection method based on the generation of pyrophosphate, byproduct of LAMP, during DNA amplification. The SERS substrate is based on a multifunctional architecture having a plasmonic core of Au nanospheres for SERS signal enhancement, which were functionalized with three different molecules: (i) a thiolated poly(ethylenglycol) that stabilizes the AuNPs in the presence of the divalent metals ions, (ii) the 1-naphthalenethiol as a Raman reporter molecule was included to trace the AuNP aggregation in relation to the SERS signal, and (iii) glutathione (GSH) that triggers the aggregation on AuNPs in presence of divalent metals ions, i.e., magnesium, which is a present in the LAMP reaction buffer, and pyrophosphate released during the DNA amplification (see Equation (1)). The design of these multifunctional AuNPs was bioinspired by the mechanisms of defense of organisms against divalent heavy metal contamination. One of these biochemical mechanisms is the synthesis of GSH due to its high affinity for divalent heavy metal ions, which allows the decrease of their toxicity, easing their elimination [37,38]. Thus, the goal of this study was to determine the possibility of combining these bioinspired AuNPs with LAMP-based DNA amplification performed in microdroplets, for the detection of *L. monocytogenes* using SERS as analytical readout. Using this approach, we were able to identify as positive, samples spiked down to 102.0 pg/µL of target DNA. The SERS signal from a sample with DNA of *Listeria innocua* was comparable with the negative control, demonstrating the specificity of this approach. Importantly, we were also able to detect target DNA in a food sample artificially inoculated with 3.6 × 10^2^ CFU/mL (i.e., 90 × 10^2^ CFU in 25 mL of ultra-high temperature (UHT) milk) of *L. monocytogenes*. 

## 2. Materials and Methods 

### 2.1. Materials 

Tetrachloroauric acid (HAuCl_4_ 3H_2_O), sodium citrate, L-glutathione reduced (GSH), 1-naphtalenethiol (1NAT), Poly(ethylene glycol) methyl ether thiol (Mw = 800 Da, TPEG), and tris(hydroxylmethyl)aminomethane (TRIS) were purchased from Sigma-Aldrich (Algés, Portugal). Magnesium sulfate and Potassium pyrophosphate were acquired from Acros Organics (Enzymatic S.A., Santo Antão do Tojal, Portugal) and Alfa Aesar (Enzymatic S.A., Santo Antão do Tojal, Portugal), respectively. All laboratory glassware was washed with aqua regia and Milli-Q^®^ ultrapure (18.2 MΩ⋅cm, Merck, Madrid, Spain) for the nanoparticle synthesis.

### 2.2. Bacterial Culture 

*L. monocytogenes* WDCM00021 and *L. innocua* WDCM00017 were selected as reference strains. The bacteria were supplied by Spanish Type Culture Collection (Parc Cientific de la Universitat de Valencia, Carrer del Catedratic Agustin Escardino Benlloch, 9, 46980 Paterna, Valencia, Spain).

### 2.3. Synthesis and Functionalization of Gold Nanoparticles (AuNPs) 

AuNPs were synthesized following a previously published protocol [39]. These citrate-coated AuNPs were then sequentially functionalized with three different thiol-bearing molecules: 1NAT, GSH, TPEG. To a 1 mL suspension of AuNP (0.25 mM Au in Milli-Q water), 0.5 µL of 1NAT solution were added (10 mM 1NAT in absolute ethanol). The sample was vortexed for 5 s, 50 µL of GSH solution were added (1 mM GSH in Milli-Q water), and vortexed again for another 5 s. Three different samples were obtained depending on the volume of TPEG solution added (1.25 mM TPEG in deionized water): *1NAT:GSH*-AuNPs (0 µL of TPEG solution), *1NAT:GSH:lowTPEG*-AuNPs (21 µL of TPEG solution), *1NAT:GSH:highTPEG*-AuNPs (27 µL of TPEG solution). The functionalized samples were then stirred at room temperature for 1.5 h. Purification of the particles was performed by centrifugation (4000 rpm, 90 min) and resuspension in 100 µL of deionized water. The samples were kept at 4 °C until use.

### 2.4. Characterization

The prepared citrate-AuNPs, and multifunctional AuNPs, were characterized through the following techniques: Dynamic Light Scattering (DLS) and Zeta Potential in a SZ-100 device (Horiba ABX SAS, Amadora, Portugal), measuring scattering at 173° in water. UV-Vis-NIR spectra of nanoparticle suspensions in water were measured in a Perkin-Elmer LAMBDA 950 spectrophotometer (Scientific Laboratory Suppliers, Wilford, Nottingham, UK). Transmission electron microscopy (TEM) of the samples was performed in a JEOL 2100 200 kV TEM (Izasa Scientific, Carnaxide, Portugal). 

### 2.5. Food Sample Analysis 

Twenty-five mL of UHT milk were mixed with 225 mL of mTA10 broth, inoculated with 10, 90 and 90 × 10^2^ CFU of *L. monocytogenes*, and homogenized for 30 s in a Stomacher 400 Circulator (Seward Limited, West Sussex, UK). The mixtures were incubated for 24 h at 35 °C. Upon completion, 1 mL was taken for DNA extraction, which was performed as previously described [40,41,42].

### 2.6. LAMP 

DNA amplification was performed by LAMP, which targeted a fragment of the *hly* gene of *L. monocytogenes*. To this end, the assay described by Garrido-Maestu et al. was selected [41]. The reactions were performed in a final volume of 25 µL composed of 15 µL of turbidometric master mix (OptiGene, Horsham, UK), 1000 nM FIP/ BIP primers, 200 nM F3/ B3 primers and 600 nM LB. Additionally, each reaction contained 4 µL of *1NAT:GSH:lowTPEG*-AuNPs and 4 µL of template DNA. The amplification was performed in a Loopamp Realtime Turbidimeter (LA-500, Eiken Chemical Co., Ltd., Tokyo, Japan) at 62 °C for 60 min. DNA extracted from *L. monocytogenes* WDCM 00021 was selected as positive control. Its concentration was determined with a NanoDrop 2000c (Thermo Fisher Scientific Inc., Waltham, MA, USA). The sequences of the primers selected were: hly-F3: TGTGTTTGAGCTAGTGGTTTGG, hly-B3: CCCATTAGGCGGAAAAGCATAT, hly-FIP: GCAGCGCTCTCTATACCAGGTACAttttAATG-TCCATGTTATGTCTCCGTTA, hly-BIP: AGGTTTGTTGTGTCAGGTAGAGCGttttCGCTTAAT-AACTGGAATAAGCCAA and hly-LB: CATCCATTGTTTTGTAGTTACAGAG.

### 2.7. Microfluidic Devices Fabrication 

The microfluidic devices for microdroplet generation, and incubation, were fabricated by conventional photo- and soft lithography methods [43,44]. Briefly, the devices were designed with AutoCAD 2013 (Autodesk SA, Barcelona, Spain), and a dark-field mask was printed (JD-Photo Data, Hitchin, UK). For the microdroplet generators, SU-8 2025 negative photoresist (MicroChem, Round Rock, TX, USA) was spin-coated onto a silicon wafer (diameter: 76.2 mm) at 500 rpm for 5 s and at 1000 rpm for 33 s. After spinning, the wafer was prebaked (3 min at 65 °C, 9 min at 95 °C). The steps mentioned above were repeated twice to obtain a final thickness close to 120 µm. Then, the coated silicon wafer was exposed to UV light through the acetate mask on a mask aligner at 750 mJ cm^−2^ (MA6BA6, Suss Microtech, Garching, Germany). Afterwards, a post-bake was performed (1 min at 65 °C and 4 min at 95 °C) and, once the wafer had cooled down, development was performed by immersing the master in SU-8 developer (PGMEA, Sigma Aldrich, Algés, Portugal) until the features were revealed. The master was hard-baked for 2 min at 170 °C and a final thickness of 120 µm was obtained, as measured by profilometry (KLA-Tencor, Milpitas, CA, USA).

For the reservoirs (traps), SU-8 2025 negative photoresist was spin-coated onto a silicon wafer (diameter: 76.2 mm) at 500 rpm for 5 s and at 1450 rpm for 40 s. After spinning, the wafer was prebaked (2 min at 65 °C, then 6 min at 95 °C). This process was repeated twice in order to obtain a final thickness close to 80 µm. Once completed, the coated silicon wafer was exposed to UV light at 500 mJ cm^−2^. After the UV exposure was performed a post-baking (1 min at 65 °C and 3 min at 95 °C) and developed with SU-8 developer (PGMEA, Sigma Aldrich, Algés, Portugal) and finally the master was hard-baked for 2 min at 170 °C. A final thickness of 80 µm was obtained, as measured by profilometry. 

For the fabrication of the polydimethylsiloxane (PDMS, Sylgard 184, Ellsworth Adhesives Iberica SA, Madrid, Spain) replicas, a mixture of PDMS pre-polymer and cross-linker (ratio 10:1, w/w) was poured on top of the master, degassed and cured for 2 h at 65 °C. The cured device was cut and peeled off from the master, and holes for tubing were made with a biopsy punch (diameter = 1.25 mm; Kai Medical, Dallas, TX, USA). Finally, for the preparation of hybrid PDMS-PDMS, PDMS replicas and flat PDMS pieces were treated with oxygen plasma for 20 s. Immediately after, the exposed surfaces of both PDMS pieces were bound. Finally, to guarantee the optimal sticking of all the features within the device, soft pressure was applied. The channels of the droplet generator devices were functionalized by flushing Aquapel^®^, followed by a fluorinated oil (HFE 7500 Novec, Sigma Aldrich, Algés, Portugal) to remove the remaining Aquapel^®^ and to avoid crystallization and channel clogging. This step is crucial to improve the hydrophobicity of the channels, to encapsulate the LAMP reaction in aqueous droplets surrounded by a carrier oil, as well as to improve the droplet stability during the process.

### 2.8. Microdroplets for LAMP-on-a-Chip 

The encapsulation of the reagents (master mix and primers, previously mentioned), NPs, and DNA samples (refer to Table 1) was made using the microdroplet generators fabricated as described above. The microdroplet generators were designed to have one inlet for the continuous phase (oil), one inlet for the dispersed phase (aqueous), and one outlet to recover the formed microdroplets (Figure 4).

First, two syringes (1 mL, Terumo) were filled with the different working solutions of the dispersed phase (LAMP Master mix + Primers + *1NAT:GSH:lowTPEG*-AuNPs + DNA samples) and of the continuous phase, (HFE 7500Novec) + Pico-Surf 1–2 % (Sphere Fluidics, Ltd., Cambridge, UK). The syringes were mounted on two syringe pumps (New Era Pump Systems), and the tubing ends were connected to the microfluidic devices. The syringe pumps were programmed for specific flow rates for the oil (Q*_continuous solution_*) of 1000 µL h^−1^, and the working solution (Q*_aqueous solution_*) of 500 µL h^−1^, causing a flow rate ratio (Q*_continuous solution_* / Q*_aqueous solution_*) of 20. 

The generated microdroplets from the outlet were recovered by an additional tubing piece, which transferred the microdroplets into the reservoirs. When the microdroplets started to fill the reservoir, a plug was inserted in the outlet of the device and the inlet tubing was burnt to make proper sealing of the reservoir. The resultant microdroplets containing the working solution were put in the oven at 62 °C for 60 min, to perform the LAMP amplification. Finally, the microdroplets in the reservoirs were used for downstream analysis in the Raman analysis.

### 2.9. SERS Measurements 

The measurements were carried out in a 300R Confocal Raman microscope (WITec GmbH, Ulm, Germany) using the 785 nm laser line (grating 600 gr cm^−1^) as the excitation source, and a 20× objective. The acquisition of different single spectra from the microdroplets in the reservoirs was performed for 1 s, and five scans per measurement were collected at a laser power of 70 mW. A minimum of five spectra were recorded for each of the samples in different droplets. The resulting SERS spectra were processed with the Project5 WITec software for baseline and cosmic ray removal corrections. Spectragryph software was used for figure preparation.

## 3. Results

### 3.1. Preparation and Characterization of Multifunctional Gold Nanoparticles (AuNPs)

#### 3.1.1. Optimization of Multifunctional AuNPs

**Figure 1:**
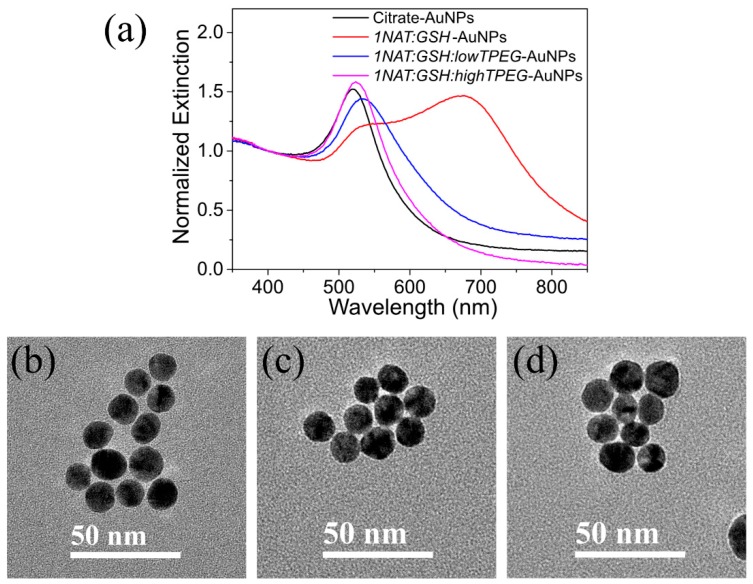
(**a**) UV-vis spectra of the multifunctional AuNPs set in comparison with initial citrate-AuNPs (black spectrum). Clearly, *1NAT:GSH:highTPEG*-AuNPs shows the highest colloidal stability, while *1NAT:GSH:lowTPEG*-AuNPs are slightly aggregated. (**b**) TEM image of 14 nm citrate-coated AuNPs used as core in the preparation of multifunctional AuNPs. Specifically, (**c**,**d**) show TEM images of *1NAT:GSH:lowTPEG*-AuNPs and *1NAT:GSH:highTPEG*-AuNPs. TEM image of *1NAT:GSH*-AuNPs is not shown as it was discarded for the subsequent experiments due to the high aggregation observed on its UV-Vis spectrum (red in (**a**)) and confirmed by DLS (Table 2).

We prepared multifunctional AuNPs in the absence of thiolated PEG, *1NAT:GSH*-AuNPs, and in presence of thiolated PEG at two initial grafting density: 16 and 20 TPEG per nm^2^, named here *1NAT:GSH:lowTPEG*-AuNPs and *1NAT:GSH:highTPEG*-AuNPs, respectively. These two grafting densities were selected because with lower initial grafting density of TPEG, the multifunctional AuNPs were not colloidally stable in presence of 8 mM of magnesium ion at pH 8.5 (data not shown), which mimics the magnesium content, and pH, of the LAMP master mix. The key role of TPEG in this system is to avoid the magnesium-conducted aggregation of AuNPs by the formation of the magnesium-GSH complex as reported previously [26]. The physicochemical characterization of these multifunctional AuNPs is summarized in Figure 1 and Table 2.

Figure 1a shows the extinction spectra of citrate and multifunctional AuNPs in water, which display different behaviors. The localized surface plasmon resonance (LSPR) of citrate-AuNPs in water is centered at λ_max_ = 520 nm, while the LSPR band of *1NAT:GSH:highTPEG*-AuNPs is red-shifted to 524 nm, which we attributed to the refractive index difference altered by the presence of TPEG on the Au surface. [45] These *1NAT:GSH:highTPEG*-AuNPs do not show any evidence of aggregation, usually resulting in a band broadening, nor do they present a new band around 600 nm in the UV-Vis spectrum (magenta spectrum in Figure 1a). However, the LSPR band of *1NAT:GSH:lowTPEG*-AuNPs not only displays a red-shift (λ_max_ = 532 nm) but also a broadening of the peak which may be explained by a slight degree of aggregation (blue spectrum in Figure 1a) [46]. In the same line, a clear aggregation trend was observed in the UV-Vis spectrum of *1NAT:GSH*-AuNPs as the intensity of the LSPR at 520 nm decreased, its λ_max_ center red-shifts and a new LSPR band appeared in the near-infrared region (red spectrum in Figure 1a). The appearance of this additional LSPR band was attributed to the coupling of the individual LSPR of the AuNPs due to the interparticle hydrogen bonding between the carboxylic groups of GSH [47]. These observations were confirmed by DLS results, as shown in Table 2. The hydrodynamic diameter of *1NAT:GSH:highTPEG*-AuNPs was 37.3 nm, which is 17.1 nm larger than citrate-AuNPs due to the presence of TPEG on the surface. Further, *1NAT:GSH*-AuNPs and *1NAT:GSH:lowTPEG*-AuNPs present a larger hydrodynamic diameter, 714 nm and 93 nm, respectively, confirming the aggregation of these multifunctional AuNPs. Zeta potential measurements (Table 2) revealed negatively charged surfaces for all AuNPs, except in the case of *1NAT:GSH*-AuNPs. The positive value of zeta potential, 20.8 mV, may be attributed to the higher formation of hydrogen bonds between the carboxylic groups of GSH due to the presence of 1NAT, a hydrophobic molecule [48,49]. Figure 1b–d shows the characteristic TEM images of the citrate-AuNPs, *1NAT:GSH:lowTPEG*-AuNPs and *1NAT:GSH:highTPEG*-AuNPs, having a spherical shape with a diameter of 14 ± 2 nm.

#### 3.1.2. Bioinspired Indirect SERS Detection

**Figure 2:**
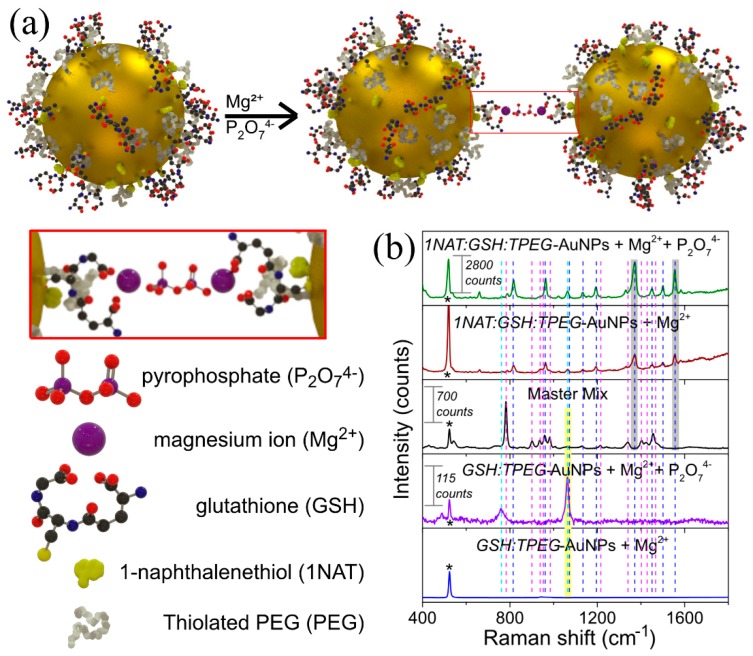
(**a**) Schematic illustration of the mechanism of detection of DNA by LAMP using an indirect SERS method. In this indirect method, the SERS signal comes from a Raman reporter molecule, 1NAT (yellow structure on AuNPs in the scheme (**a**)) in this manuscript. Thus, the SERS signal of 1NAT is directly proportional to the 14 nm-AuNPs aggregation triggered by the complexation of the carboxylic group of GSH (molecular structure with red, black, blue and yellow atoms represent oxygen, carbon, nitrogen and sulfur, respectively) that is attached on Au surface and pyrophosphate (molecular structure which purple and red atoms represent phosphor and oxygen, respectively) generated during LAMP reaction with magnesium ions (purple sphere in the scheme). In LAMP, the pyrophosphate ions are produced during DNA amplification because pyrophosphate is released from dNTP as a by-product. In the design of multifunctional AuNPs thiolated poly(ethylene glycol) (TPEG; white molecule in the scheme) was included to avoid the magnesium-related aggregation, increasing the colloidal stability of *1NAT:GSH*-functionalized AuNPs in presence of magnesium ions. Illustration inside the red rectangle represents a zoom-in of the Au-GSH:Mg:P_2_O_7_:Mg:GSH-Au complex formed. (**b**) Aggregation behavior of AuNPs functionalized either *GSH:TPEG* or *1NAT:GSH:TPEG* at 5 mM TRIS buffer pH 8.5 and 8 mM Mg^2+^ and in absence or presence of potassium pyrophosphate (P_2_O_7_^4−^) at concentration of 6 µM. The SERS spectra in presence of pyrophosphate show clearly the either 1NAT (green spectrum) or GSH (purple spectrum) characteristic peaks due to the formation of AuNPs aggregates triggered by the complexation of GSH-attached AuNPs (0.32 mM of Au) with Mg^2+^ and pyrophosphate ions. This aggregation improved the resonance of LSPR with the selected 785 nm laser line. In absence of pyrophosphate ions, the SERS signal was either null for GSH or lower for 1NAT due to the AuNPs being stable in presence of magnesium ions (blue and dark red spectra, respectively). Despite both types of multifunctional AuNPs work using this SERS strategy, the high Raman signal (back spectrum) acquired from the master mix used in LAMP (mainly from dNTPs) interferes enormously when GSH is used as SERS reporter. Therefore, multifunctional AuNPs that contain 1NAT were selected due to their higher Raman cross section and the fact that one of the SERS band (1368 and 1553 cm^−1^ ring stretching) does not overlap with any master mix or PDMS (microfluidic device, see Figure 3b) bands. Characteristic peaks for GSH and 1NAT that do not overlap with the master mix Raman peaks which are labeled with yellow and gray shaded areas, respectively. Dashed lines highlight the position of the Raman peaks corresponding to dNTPs of the master mix (magenta dashed line) and SERS peaks of GSH (cyan dashed line) and 1NAT (blue dashed line). * indicates the Raman peak from the silicon wafer used as substrate for these measurements (520 cm^−1^).

We proposed a bioinspired indirect detection based on the quantity of pyrophosphate ions generated during LAMP by using an indirect SERS detection strategy using a Raman reporter. Figure 2a schematically describes the SERS substrate architecture designed for this purpose. The detailed synthetic procedures are described in the Materials and Methods section. Briefly, three thiolated molecules, GSH, 1NAT and TPEG, with different functionalities were covalently bonded to monodisperse 14 nm AuNPs via a Au-S bond, which acts as SERS optical enhancer [50]. These thiolated molecules were strategically selected as GSH is a chelating agent of divalent metal ions [37], and TPEG molecules act as a stabilizing agent against magnesium ion-induced aggregation, as previously reported [26]. Thus, the detection of the DNA amplification was based on the aggregation of *1NAT:GSH:TPEG*-AuNPs triggered by the complexation of AuNPs via the carboxylic group of GSH and pyrophosphate released by the incorporation of dNTPs into the DNA strand during polymerization with free magnesium ions (see scheme on Figure 2a). The aggregation of AuNPs in the presence of pyrophosphate could be measured in terms of the SERS signal related to 1NAT, the Raman reporter selected in this study. The variation in the SERS signal of 1NAT was proportional to the degree of AuNPs aggregation, generating a high density of hot spots, and promoting a better resonance with 785 nm laser line [33], which enhanced the SERS signal of 1NAT that can be directly related to the amount of pyrophosphate ions generated in the LAMP reaction (Figure 2b). In order to analyze the advantages of using 1NAT as a Raman reporter instead of directly detecting GSH, we prepared two different batches of AuNPs either *GSH:TPEG* (ratio 60:4 molecules per nm^2^) or with *1NAT:GSH:lowTPEG* (ratio 6:30:16 molecules per nm^2^), which were subsequently incubated with Mg^2+^ (8 mM) and Mg^2+^/pyrophosphate (8 mM/6 µM) at 62 °C for 60 min, mimicking the LAMP reaction conditions. In the presence of pyrophosphate ions, the SERS spectra of 1NAT and GSH were clearly recorded (see green and purple spectra in Figure 2b), demonstrating that the formation of a stable complex between GSH, magnesium, and pyrophosphate triggered the aggregation of the NPs. The obtained SERS spectra are dominated by ring stretching (1553, 1503, and 1368 cm^−1^), C-H bending (1197 cm^−1^), ring breathing (968 and 822 cm^−1^), ring deformation (792, 664, 539 and 517 cm^−1^), and C-S stretching (389 cm^−1^) for 1NAT (Figure 2b green spectrum) [51], and C-N stretching (1051 cm^−1^) and Amide V (791 cm^−1^) for GSH (Figure 2b purple spectrum) [52]. In the absence of pyrophosphate, the SERS spectrum of GSH was not present (Figure 2b blue spectrum), while the SERS spectrum of 1NAT was observed, though displaying low intensity (Figure 2b red spectrum). The latter may be explained by the lack of hot spots as the multifunctional AuNPs remained stable, in turn avoiding the enhancement of the SERS signal. This feature demonstrated the importance of the presence of TPEG on the Au surface because TPEG molecules prevented the magnesium-trigged aggregation of carboxyl-functionalized AuNPs, as reported previously [25]. Figure 2b also shows the Raman spectrum of commercially available turbidimetric master mix (black spectrum in the Figure 2b), an indispensable reagent for LAMP reaction. This spectrum corresponds to deoxyribonucleotide triphosphate (dNTPs), which releases pyrophosphate during DNA polymerization [23,24]. The Raman spectrum is dominated by the stretching vibration of the three-phosphate group (1135 cm^−1^), C-C and C-N stretching vibrations involving aromatic rings (1344 and 1458 cm^−1^), 5C-H and 6CH-bendings (1214 and 1245 cm^−1^), and cytosine vibration (788 cm^−1^) [53,54,55]. This matrix background interfered in the direct detection of GSH due to the very low Raman cross-section of this molecule and the peaks being overlapped by the LAMP matrix. In this sense, the stronger Raman cross-section of 1NAT offers was of vital importance. It is also worth highlighting that some of the characteristic peaks of 1NAT do not overlap with the characteristic peaks of the master mix, allowing the indirect SERS detection of the LAMP reaction (see peaks highlighted with shaded gray area in Figure 2b).

#### 3.1.3. SERS Monitoring of LAMP with 1NAT:GSH:TPEG-AuNPs

**Figure 3:**
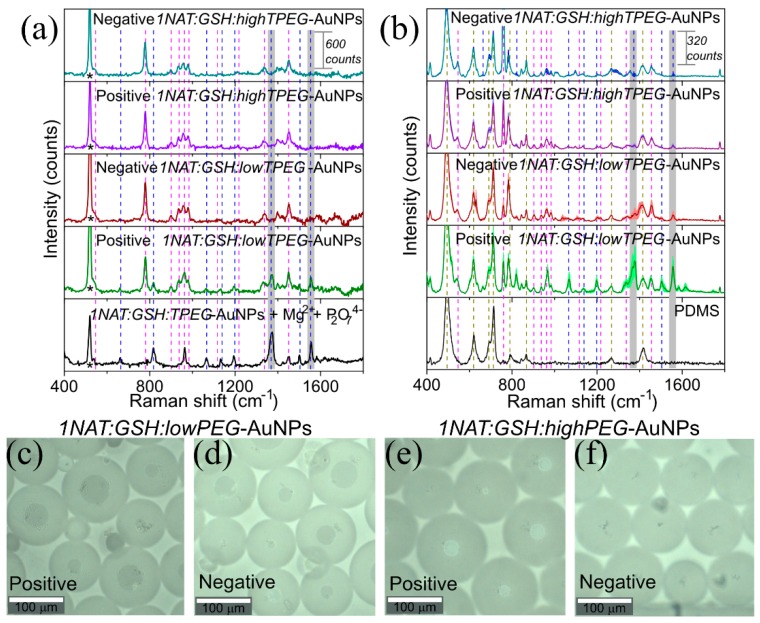
Detection of LAMP by SERS derived from AuNPs aggregation due to the pyrophosphate generation. Briefly, target DNA extracted from *L. monocytogenes* was amplified in the presence of either *1NAT:GSH:lowTPEG*-AuNPs or *1NAT:GSH:highTPEG*-AuNPs by off-chip LAMP at 62 °C for 60 min. In the negative control, sterilized water was added instead of target DNA. A positive reaction was indicated by the enhancement of SERS signal of 1NAT (the Raman reporter used in this manuscript) due to the aggregation of multifunctional-AuNPs triggered by the complexation of GSH presented on Au surface and pyrophosphate ions that is a byproduct of DNA amplification, with free Mg^2+^. The SERS analysis was carried out (**a**) off-chip and (**b**) within the formed microdroplets. Dashed lines highlight the position of the Raman peaks corresponding to PDMS (dark yellow dashed line) and dNTPs of the master mix (magenta dashed line) and SERS peaks of 1NAT (blue dashed line). Gray shaded areas indicate the selected characteristic peaks of 1NAT (1368 and 1553 cm^−1^, ring stretching) to follow the variation of SERS signal respect with the generation of pyrophosphate ions during the DNA amplification. Shadowed areas in the spectra of (**b**) represent the standard error of the mean (SEM) of 6–13 SERS spectra. * in (**a**) indicates the Raman peak from silicon wafer (520 cm^−1^). The best results were obtained with *1NAT:GSH:lowTPEG*-AuNPs. Representative bright field images of microdroplets formed during the encapsulation of amplification product of LAMP assays obtained in (**c**)**,** (**e**) positive and (**d**)**,** (**f**) negative controls in presence of *1NAT:GSH:lowTPEG*-AuNPs *1NAT:GSH:highTPEG*-AuNPs, respectively.

We explored the applicability of these *1NAT:GSH:lowTPEG*-AuNPs and *1NAT:GSH:highTPEG*-AuNPs for the monitoring the LAMP reaction. For that, we performed a complete assay for the differentiation between a negative sample (in the absence of the target DNA) and a positive sample containing the target DNA sample from a *L. monocytogenes* extract at a concentration of 10.2 ng/µL. As a first approximation, the LAMP reaction was performed off-chip in Eppendorf tubes following the conventional approach. Both multifunctional AuNPs containing low and high TPEG were tested. Figure 3a shows the LAMP results via SERS, and as expected, the Raman characteristic peaks from the LAMP master mix could be observed in each average spectrum. The characteristic peaks of 1NAT were clearly identified only in the positive sample of *1NAT:GSH:lowTPEG*-AuNPs (see gree spectrum in Figure 3a). Further, no differences were observed between the negative samples (i.e., not target DNA) in both AuNPs types and the positive (i.e., presence of target DNA) of *1NAT:GSH:highTPEG*-AuNPs. Thus, the aggregation of AuNPs triggered by the complexation of GSH and pyrophosphate ions with free magnesium ions showed higher efficiency with AuNPs containing a low number of TPEG molecules. This means that an excess of TPEG molecules on the Au surface can prevent the co-complexation between GSH and pyrophosphate with magnesium ions likely due to the change of TPEG conformation from mushroom to brush [56].

### 3.2. LAMP on-a-Chip SERS Detection

#### 3.2.1. LAMP on-a-Chip Setup

**Figure 4:**
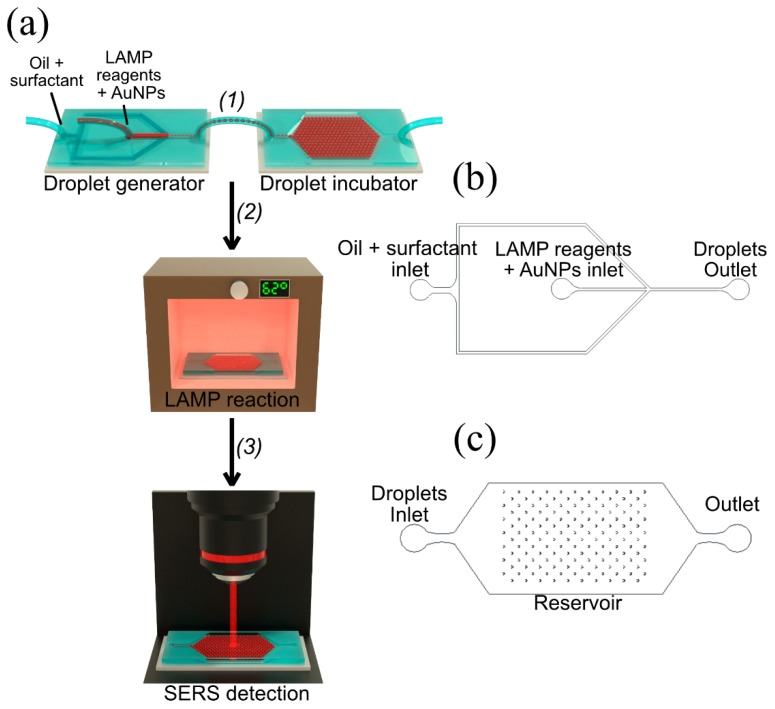
(**a**) Schematic illustration of the experimental setup for microdroplet array-based SERS LAMP analysis: (1) Microdroplet generator device; (2) Microdroplets stored into trapping array were incubated at 62 °C for 60 min to produce the DNA amplification by LAMP and (3) SERS signal emerging from positive droplets was detected on the microdroplets contained in the reservoir using a confocal Raman microscope; (**b**) AutoCAD design for the microdroplets generator and (**c**) AutoCAD design of the microdroplet incubator. The size of microdroplets generated was ≈ 100 µm.

The experimental process of the microdroplet-based SERS LAMP analysis is schematically illustrated in Figure 4. The LAMP-on-a-chip process involves a droplet generator device coupled to a microdroplet trapping reservoir (Schematic illustration in Figure 4a and AutoCAD designs in Figure 4b,c, respectively). Both devices were fabricated using a PDMS replica for the device features, as well as a blank PDMS substrate to seal the fluidic channels (PDMS/PDMS as described in the Materials and Methods section). 

Before testing the LAMP reaction inside the droplets, we investigated the efficiency of the droplet formation in the presence of the mixture of the LAMP components plus the aggregated AuNPs as per complexation of GSH-Mg-pyrophosphate. For that purpose, an off-chip LAMP reaction was performed in microtubes at 62 °C for 60 min. A volume of 25 µL of the mixture was then encapsulated into aqueous droplets using the flow focusing device (see Figure 4b) [57]. Then, microdroplets were hydrodynamically trapped into reservoirs and bright field images were obtained using a confocal Raman with a 20× objective (Figure 3c–f). The average size of the microdroplets was 107 µm with a coefficient of variation of 15.8% (300 droplets were measured with ImageJ). This coefficient of variation was higher than what was expected for microdroplets using a flow-focusing geometry, however it could be explained by the coalescence of some of the droplets in the reservoir. 

The results of the SERS analysis using a 785 nm laser excitation wavelength in the microdroplets are summarized in Figure 3b. Figure 3b shows the Raman spectrum of PDMS, the material of the reservoir. The spectrum is dominated by Si-O-Si symmetric stretching (488 cm^−1^), Si-CH_3_ symmetric rocking (687 cm^−1^), Si-C symmetric stretching (708 cm^−1^), CH_3_ asymmetric rocking + Si-C asymmetric stretching (787 cm^−1^), CH_3_ symmetric rocking (862 cm^−1^), CH_3_ symmetric bending (1262 cm^−1^), and CH_3_ asymmetric bending (1412 cm^−1^) [58]. The acquired spectra of the microdroplets contents resulted in a mixture of that of the multifunctional AuNPs, the master mix and the PDMS, as expected. Most importantly, the selected characteristic peaks of 1NAT (1368 and 1553 cm^−1^) did not overlap with the characteristic peaks of PDMS, allowing the identification of positive and negative samples (shaded area in Figure 3b).

The analysis of the positive and negative samples using *1NAT:GSH:lowTPEG*-AuNPs and *1NAT:GSH:highTPEG*-AuNPs inside of the microdroplets confirmed the acquired results off-chip as expected: the best differentiation between positive and negative control was obtained in the presence of *1NAT:GSH:lowTPEG*-AuNPs due to higher yield of aggregation between these AuNPs and magnesium pyrophosphate crystals.

#### 3.2.2. LAMP on-a-Chip for DNA Extracted from *L. monocytogenes* Culture

**Figure 5:**
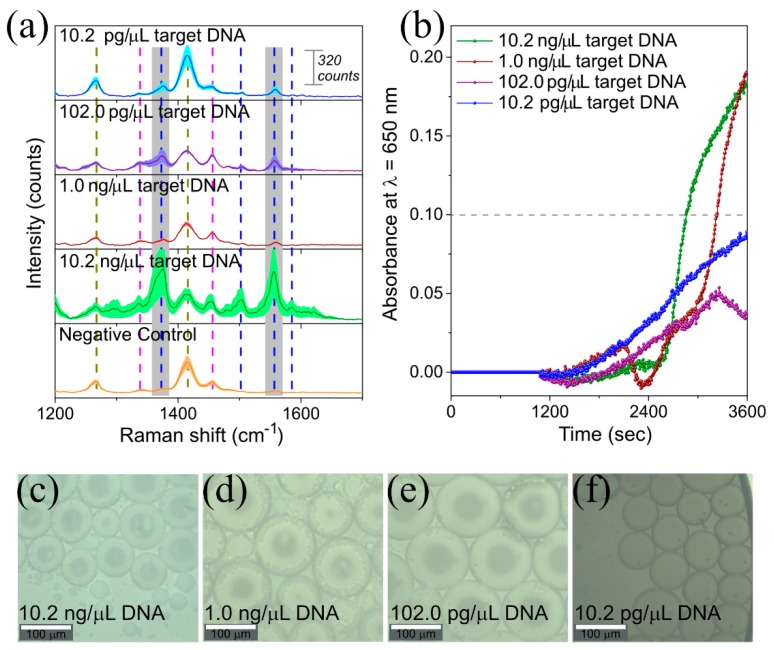
(**a**) Comparison of LAMP-on-a-chip SERS detection with (**b**) LAMP real-time turbidity detection for DNA extracted from *L. monocytogenes.* LAMP reaction was performed at 62 °C for 60 min with different target DNA concentrations. In the negative control, sterilized water was added instead of extracted DNA. A positive reaction was indicated by the enhancement of SERS signal of 1NAT (i.e., the Raman reporter used in this work) due to the aggregation of *1NAT:GSH:lowTPEG*-AuNPs triggered by the complexation of GSH presented on Au surface and pyrophosphate ions that is a byproduct of DNA amplification, with free Mg^2+^ in the case of (**a**) and by increasing of absorbance at 650 nm due to formation of magnesium pyrophosphate crystals in (**b**). In the graph shown in (**a**), dashed lines highlight the position of the Raman peaks corresponding to PDMS (dark yellow dashed line) and dNTPs of the master mix (magenta dashed line) and SERS peaks of 1NAT (blue dashed line). Gray shaded areas in (**a**) indicate the selected characteristic peaks of 1NAT (1368 and 1553 cm^−1^, ring stretching) to follow the variation of SERS signal respect to the generation of pyrophosphate ions during DNA amplification (see Figure 6). Shadowed area in the spectrum represents the standard error of the mean (SEM) of 6–13 SERS spectra. Representative bright field images of stored microdroplets into trapping array are shown after target DNA amplification by LAMP-on-a-chip at a concentration of (**c**) 10.2 ng/µL (**e**) 1.0 ng/µL, (**d**) 102.0 pg/µL and (**f**) 10.2 pg/µL.

**Figure 6:**
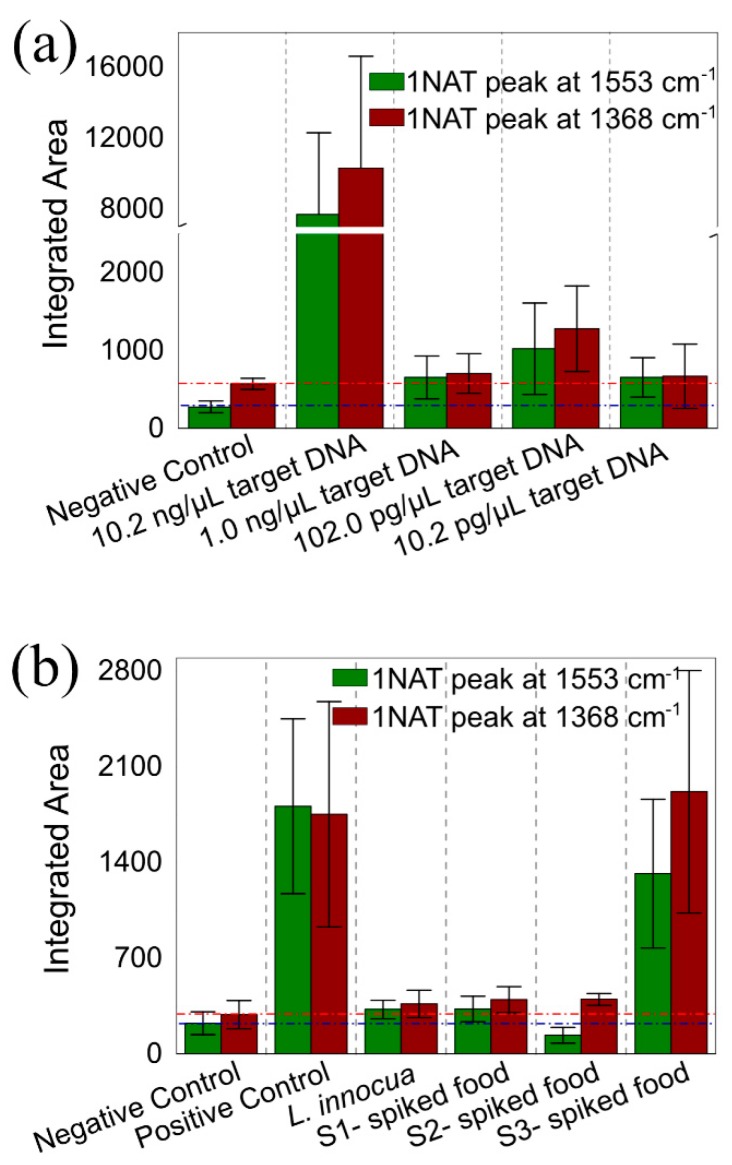
LAMP-on-a-chip SERS assay were performed for *L. monocytogenes* detection in the conditions presented in (**a**) Figure 5 and (**b**) Figure 7. The bar charts show the integrated area of 1NAT peaks centered at 1553 and 1368 cm^−1^ (shaded area in Figure 5 and Figure 7) at (**a**) each of the target DNA concentrations, and these are compared to a negative control sample (i.e., no target DNA present); and (**b**) of the target DNA extracted from UHT milk sample that was inoculated with 10 (S1-spiked food), 90 (S2-spiked food), and 90 × 10^2^ (S3-spiked food) CFU of *L. monocytogenes*, these are compared to a positive control (i.e., 10.2 ng/µL of target DNA), a negative control (i.e., no target DNA present) and a control sample with DNA from *L. innocua* instead of the target DNA. Integrated areas were obtained by acquiring 6–13 SERS spectra inside the microdroplets and the error bars represent one standard error of the mean (SEM). The dashed lines give visual clarification of the integrated areas of the negative control.

To study the sensitivity of this method, we performed the LAMP on-a-chip amplification and indirect SERS detection with different concentrations of target DNA (Table 1), subsequently comparing these results with off-chip LAMP real-time turbidity detection (Figure 5 and Figure 6a). Ten-fold serial dilutions of DNA extracted from *L. monocytogenes* were used as target (initial [DNA] = 10.2 ng/µL, down to 1/1000). All solutions, namely target DNA dilution, LAMP reagents, and oil pre-mixed with a surfactant (2 % w/v), were freshly prepared before being injected into the microfluidic chip. Afterwards, DNA target molecules, LAMP reagents and multifunctional *1NAT:GSH:lowTPEG*-AuNPs were encapsulated into water-in-oil-microdroplets, and subsequently stored in the reservoirs. DNA target molecules were amplified during the LAMP process by placing the reservoirs in an oven at 62 °C for 60 min. Finally, the SERS signal of 1NAT from seven to thirteen droplets in each reaction was recorded using the confocal Raman microscope (see scheme of Figure 4a). The average size of formed microdroplets was 85 µm with a coefficient of variation of 33% (Figure 5c–f, 350 droplets were measured with ImageJ). The smaller size observed and the higher coefficient of variation can be explained by the possible coalescence of some of the droplets as well as by the evaporation of droplet contents during the heating step. Figure 5a,b show the results of LAMP-on-a-chip SERS detection and LAMP real-time turbidity detection, respectively. Figure 5a shows a close-up of the spectral range in which the selected characteristic 1NAT peaks (1368 and 1553 cm^−1^) were identified, and in which the shaded area around the solid line is the standard error of the mean (SEM). The 1NAT peaks were clearly identified in the average SERS spectrum of 10.2 ng/µL target DNA (green spectrum in Figure 5a). To improve the analysis of the acquired data from each sample, the area of each selected 1NAT peak was integrated and is presented in Figure 6a. Positive samples were discriminated from the negative control down to a DNA target concentration of 102.0 pg/µL (Figure 6a), while using the turbidity method the minimum DNA concentration detected was 1.0 ng/µL, considering as positive the samples that present an absorbance ≥ 0.1 (Figure 5b).

#### 3.2.3. LAMP on-a-Chip for DNA Extracted from Food Products

**Figure 7:**
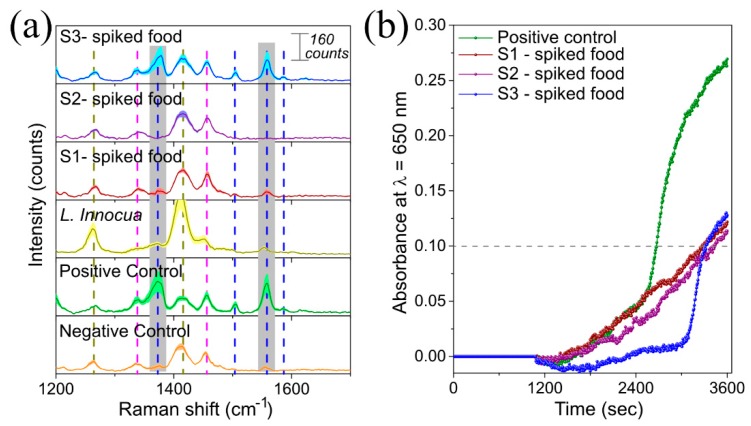
Ability of LAMP-on-a-chip SERS assay to detect *L. monocytogenes* in UHT milk. Briefly, 25 mL of UHT milk were inoculated with 10 (S1-spiked food), 90 (S2-spiked food), and 90 × 10^2^ (S3-spiked food) CFU of *L. monocytogenes* (Table 1). Then, the target DNA was extracted and analyzed by LAMP. The detection was carried out by (**a**) SERS (i.e., using LAMP-on-a-chip SERS assay) and (**b**) turbidity (e.i. LAMP real-time turbidity detection) as reference. Both LAMP reactions were performed in the presence of *1NAT:GSH:lowTPEG*-AuNPs at 62 °C for 60 min. Positive control consists of pure target DNA extracted from *L. monocytogenes* at concentration of 10.2 µg/µL. In the negative control, sterilized water, and DNA extracted from *L. innocua* were added instead of target DNA. A positive reaction (i.e., detection of *L. monocytogenes*) was indicated by the enhancement of SERS signal of 1NAT due to the AuNPs aggregation triggered by the complexation of GSH via its carboxylic groups and pyrophosphate generated as byproduct of DNA amplification with free Mg^2+^ in the case of (**a**), and by increasing of the absorbance at 650 nm due to the formation of magnesium pyrophosphate crystals (possible interferences from the presence of AuNPs) in (**b**). In the graph shown in (**a**), dashed lines highlight the position of the Raman peaks corresponding to PDMS (dark yellow dashed line) and dNTPs of the master mix (magenta dashed line) and SERS peaks of 1NAT (blue dashed line). Gray shaded areas in (**a**) indicate the selected characteristic peaks of 1NAT (1368 and 1553 cm^−1^, ring stretching) to follow the variation of SERS signal respect to the generation of pyrophosphate ions during the DNA amplification (see Figure 6). Shadowed area in the spectrum represents the standard error of the mean (SEM) of 6–13 SERS spectra.

To validate the new sensing strategy in a real setting, a proof-of-concept experiment demonstrated specific detection of *L. monocytogenes* DNA extracted from inoculated UHT milk. For this purpose, 10, 90, and 90 × 10^2^ CFU of *L. monocytogenes* were spiked in 25 mL of UHT milk (See Table 1 and labels in Figure 6b and Figure 7 as S1-, S2-, and S3-spiked food, respectively). Then, DNA extraction was performed from 1 mL of inoculated food and subsequently 4 µL of extracted DNA were amplified using the LAMP on-a-chip protocol. In addition, to study the selectivity of the system, we performed the LAMP on-a-chip reaction using DNA extracted from *L. innocua* culture as target DNA (see Table 1). Figure 7a shows a close-up of the spectral range in which can identify the selected characteristic 1NAT peaks (1368 and 1553 cm^−1^), and in which the shadowed area around the solid line indicates the standard error of the mean (SEM). The 1NAT peaks could only be clearly identified in the average SERS spectrum of S3-spiked food (green spectrum in Figure 7a). To improve the analysis of the acquired data from each sample, the area of each selected 1NAT peak was integrated and is shown in Figure 6b. Figure 6b shows that the assay was specific enough to discriminate between *L. monocytogenes* and *L. innocua*, since no SERS signal of 1NAT could be observed in the *L. innocua* spectrum (yellow spectrum in Figure 7a), while the SERS signal of 1NAT was clearly identified in the positive control (green spectrum in Figure 7a). Moreover, Figure 6b shows that the extracted DNA from milk could be clearly detected in the sample that was inoculated with 90 × 10^2^ CFU (S3-spiked food in Figure 6b and Figure 7), while SERS signals from samples with 10 (S1-spiked food in Figure 6b and Figure 7) and 90 (S2-spiked food in Figure 6) CFU of *L. monocytogenes* had a comparable integrated area to that of the negative control. The latter is in agreement with the results obtained with real time LAMP turbidity assay (Figure 7b). However, the identification of this sample as positive was clearer with the LAMP-on-a-chip SERS detection, as the absorbance at 650 nm of this sample, S3-spiked food, barely performed above the threshold value of absorbance 0.1 (Figure 7b). The presence of *L. monocytogenes* in the samples with lower concentration could not be confirmed neither by SERS nor by turbidity. This suggests the potentiality of the LAMP-SERS technology for foodborne pathogens detection in real matrices. 

## 4. Discussion

A SERS bioinspired substrate was designed based on the mechanism of defense of organisms against heavy metal contamination. Such biochemical mechanism of defense is based on the synthesis of small molecules, such as GSH with high affinity for heavy metal ions that can chemically bind them to decrease their toxicity favoring their elimination. GSH is included in these biosynthesized small molecules and therefore it was selected to functionalize a SERS substrate based on AuNPs [37,38]. In the methodology presented here, we took advantage of two components of the LAMP reaction: magnesium (Mg^2+^) and pyrophosphate (P_2_O_7_^4−^) ions. Magnesium is an antioxidant agent, necessary for the *de novo* synthesis of GSH and as a divalent metal ion has high affinity for the complexation via carboxylic group [59]. Thus, the aggregation of GSH-AuNPs may occur in the presence of Mg^2+^ by formation of a stable complex between of carboxylic groups of GSH and free magnesium ions. The chelating capacity of GHS has been used to design bioinspired sensors for heavy metals detection such as cadmium (II) [37,60] and lead (II) [37,61]. However, this simple interaction was not enough to develop a detection method for nucleic acids via LAMP due to magnesium concentration being constant in the reaction mixture. For this reason, an indirect detection of DNA amplification was selected. This indirect detection was based on the generation of pyrophosphate by incorporation of dNTPs into the DNA strand during polymerization [23]. Thus, the SERS substrate was co-functionalized with thiolated PEG to stabilize AuNPs in presence of magnesium ions, having a positive effect on the behavior of the AuNPs towards their controlled aggregation. Despite the existence of a commercially available instrument to monitor LAMP via the increase of turbidity, related to the formation of magnesium pyrophosphate crystals, those turbidity measurements cannot be automated or miniaturized [23]. In the present work, the presence of pyrophosphate ions triggered the aggregation of *GHS:TPEG*-functionalized AuNPs by co-complexation of carboxylic group of GSH and pyrophosphate ions with Mg^2+^ [26]. After confirming that the signal of the GSH was not intense enough due to its low Raman-cross section, a Raman reporter, 1NAT was included in the formulation of these multifunctional AuNPs. This three-molecule architecture approach allowed the control of the process at throughout the required levels of the process. In this way, the GSH could chelate the magnesium ions, while having enough stability balanced by the presence of the TPEG molecules on the surface, and finally the 1NAT allowed the indirect SERS detection of the presence of amplified pathogen DNA. Furthermore, the use of a modular microfluidic approach towards the miniaturization of analytical methods is increasingly important in the food industry [62]. In this case, the use of microdroplets was aligned with the most relevant application of microdroplets technology on the market, ddPCR. The use of LAMP eases the integration process in microfluidic devices as no temperature cycles are needed, widening the possibilities of expansion of the technology such as the use of novel materials in microfluidics for scale-up. The integration of these advanced techniques was tested through a LAMP-on-a-chip SERS detection for *L. monocytogenes* in UHT milk. This LAMP-SERS approach demonstrated a higher sensitivity than the conventional LAMP-turbidity detection (102.0 pg/µL vs 1.0 ng/µL of target DNA). In the case of food samples, it was possible to discriminate as positive a UHT milk sample after enrichment, with a starting concentration of 3.6 × 10^2^ CFU/mL (i.e., 90 × 10^2^ CFU in 25 mL of UHT milk) of *L. monocytogenes.* Compared with conventional technologies for LAMP-amplified product detection, this LAMP-in-microdroplets SERS approach could eliminate fluorescent dye labeling steps and enhance the sensitivity. In addition, this SERS detection strategy potentially offers an alternative method for nucleic acid detection by monitoring the amount of pyrophosphate generated in different nucleic acid amplification reactions catalyzed by DNA or RNA polymerases. Droplet-based approaches for LAMP have already been explored in either micro-well, droplet array and even microdroplets fashion [63,64,65,66]. Ma et al. [30] executed LAMP for vancomycin-resistant *Enterococcus* DNA detection across 240 trapped and separated microdroplets, which contained only a volume of 0.2 nL, after only 40 min of reaction at 56 °C. These approaches are all moving towards digital LAMP technology, in the same way that PCR evolved to ddPCR in the past decade. The shift is expected as it offers quantification possibilities, automation, and miniaturization. Microdroplets have a more superior automation and miniaturization capacity than micro-well based approaches, as well as offering a much higher throughput during the sample processing and the downstream analysis. Besides this, using microdroplets combined with SERS increases the potentiality of this approach, avoiding photobleaching and the use of fluorescence labels, and more importantly enables multiplexing. Microdroplets create an isolated environment in which the local concentration of NPs increases close to the bacteria, or targeted organisms. In this way, it will be possible to create much more efficient detection protocols when compared to bulk or even millidroplet based approaches.

## Figures and Tables

**Table 1:** Samples analyzed by LAMP-on-a-chip SERS detection.

Sample Label	Description
Negative Control	Water added instead of target DNA in the LAMP reaction
Positive Control	10.2 ng/µL target DNA from *L. monocytogenes* in the LAMP reaction
10.2 ng/µL target DNA	10.2 ng/µL target DNA from *L. monocytogenes* in the LAMP reaction
1.0 ng/µL target DNA	1.0 ng/µL target DNA from *L. monocytogenes* in the LAMP reaction
102.0 pg/µL target DNA	102.0 pg/µL target DNA from *L. monocytogenes* in the LAMP reaction
10.2 pg/µL target DNA	10.2 pg/µL target DNA from *L. monocytogenes* in the LAMP reaction
*L. innocua*	13.4 ng/µL DNA from *L. innocua* in the LAMP reaction
S1 – spiked food	DNA extracted from 25 mL UHT milk inoculated with 10 CFU of *L. monocytogenes*
S2 – spiked food	DNA extracted from 25 mL UHT milk inoculated with 90 CFU of *L. monocytogenes*
S3 – spiked food	DNA extracted from 25 mL UHT milk inoculated with 90 × 10^2^ CFU of *L. monocytogenes*

**Table 2:** Physicochemical Characterization of the multifunctional AuNPs set.

	Citrate-AuNPs	*1NAT:GSH*-AuNPs	*1NAT:GSH:lowTPEG*-AuNPs	*1NAT:GSH:highTPEG*-AuNPs
Hydrodynamic diameter ^1^ (nm)	20.2 ± 0.1	714.6 ± 75.9	92.6 ± 8.5	37.3 ± 2.2
PDI ^2^ (%)	33 ± 2	106 ± 15	42 ± 2	35 ± 16
Z potential ^3^ (mV)	69.1 ± 1.8	20.8 ± 1.9	15.3 ± 4.8	30.6 ± 1.0

^1^ Mean hydrodynamic diameter and polydispersity index. ^2^ obtained by DLS at a scattering angle of 173° and 25 °C. DLS measurements were carried by triplicate: mean ± standard deviation (SD). ^3^ Zeta potentials were measured in 3 runs (mean ± SD).
